# Determinants of exclusive breastfeeding among mothers in Ghana: a cross-sectional study

**DOI:** 10.1186/1746-4358-8-13

**Published:** 2013-10-14

**Authors:** Anthony Mwinilanaa Tampah-Naah, Akwasi Kumi-Kyereme

**Affiliations:** 1 Department of Environment and Resource Studies, University for Development Studies, Wa Campus, Wa, Ghana; 2 Department of Population and Health, University of Cape Coast, Cape Coast, Ghana

**Keywords:** Exclusive breastfeeding, Mothers, Infants, Ghana

## Abstract

**Background:**

Mothers are encouraged to practice exclusive breastfeeding for the first six months of a child’s life. The general objective of the study was to assess the predictors of exclusive breastfeeding among mothers in Ghana.

**Methods:**

This was a cross-sectional study using data from the 2008 Ghana Demographic and Health Survey (GDHS). The study sample (n = 316) was based on infants (0–5 months old) during the survey period; extracted from the individual (women) data set. Binary logistic regression was used to examine the association between selected independent variables.

**Results:**

In general, the rate of exclusive breastfeeding was 64 percent. Marital status, region and place of delivery were found to be associated with the practice of exclusive breastfeeding. The logistic regression model illustrated mothers from the Volta Region, those who delivered in a government health facility, and mothers who perceived their infants to be average in size were more likely to practice exclusive breastfeeding.

**Conclusion:**

Appropriate health education programmes by the Ministry of Health targeting mothers who are less likely to practice exclusive breastfeeding are recommended.

## Background

The practice of exclusive breastfeeding in some countries in sub-Saharan Africa is undesirable in comparison to the optimum period of six months set forth by WHO and United Nation Children’s Fund (UNICEF) [1]. Some countries with low practice of exclusive breastfeeding rates include Chad (2%), Cote d’Ivoire (4%), Gabon (6%), and Sierra Leone (8%) [2]. Other countries in the region which have achieved high levels of exclusive breastfeeding include Benin (70%), and Rwanda (85%) [2,3]. Ghana’s exclusive breastfeeding rate is about 63% [4]. Although there is a steady progress in the rates of exclusive breastfeeding in the sub-region, it implies that social and environmental factors may be influencing such rates. These have challenged some sub-regional governments to adopt, formulate, and implement strategies to improve the practice of exclusive breastfeeding among mothers.

To ensure that infants are exclusively breastfed, a number of interventions have been implemented in Ghana. Some of these interventions include the adoption of the 1991 Baby-Friendly Hospital Initiative (BFHI), and the formation of BFHI Authority. The BFHI Authority was to oversee the implementation of the initiative, development of a breastfeeding policy, and strategies to guide implementation of the BFHI. The BFHI Authority was also to ensure the training of master-trainers in lactation management for all hospitals in the country. The use of health workers provided mothers with early support for exclusive breastfeeding. However, obstacles such as family pressures tend to hinder the practice of exclusive breastfeeding [5].

Another intervention has been the Ghana Breastfeeding Promotion Regulation 2000 (otherwise known as Legislative Instrument [LI] 1667). The main aim of the LI 1667 was to prevent aggressive marketing of breast milk substitutes, and thus promote breastfeeding in the country [6]. According to the Food and Drugs Board of Ghana [7], the unsuccessful campaign to promote exclusive breastfeeding in the country is due to the violation of Ghana’s Breastfeeding Promotion Regulation 2000 on the marketing of breast milk substitutes.

Also, other interventions including Information, Education and Communication (IEC) materials, and advocacy materials are designed for use by health professionals, and for the general public. Formulation and implementation of breastfeeding interventions are essential to increase exclusive breastfeeding. However, maternal and child factors play a paramount role. The study, therefore, sought to examine some likely factors that predict the practice of exclusive breastfeeding among mothers in Ghana.

## Methods

The practice of exclusive breastfeeding for research purposes is either defined based on detailed history of feeding in the past 24-hours preceding a survey of infants (0–6 months old) or based on a recall period of feeding history of an infant (0–6 months old) since birth. The study data was extracted from the 2008 Ghana Demographic and Health Survey (GDHS); specifically, the individual (women) data set. To validate the data, the rate of exclusive breastfeeding was recalculated using the ‘24-hours’ definition, as used in the 2008 GDHS. The ‘24-hours’ period of exclusive breastfeeding is defined by WHO as a mother or caregiver giving nothing else but breast milk in the last 24 hours preceding an interview or a survey [8].

The GDHS are national-level population and health surveys conducted in Ghana as part of the global Demographic and Health Survey programme. They are conducted every five years. The first survey was conducted in 1988. All the surveys are implemented by the Ghana Statistical Service (GSS) in collaboration with the Ghana Health Service to obtain detailed information including but not limited to maternal and child health [4].

The 2008 GDHS used a two-stage sample design to allow for separate estimates of key indicators for each of the 10 regions in Ghana, as well as for urban and rural areas separately. The first stage involved selecting sample clusters from the 2000 Population and Housing Census. The master sampling frame consisted of 412 clusters which were selected using systematic sampling with probability proportional to size. The second stage of the design involved the systematic sampling of 30 households listed in each cluster. Women age 15–49 and men age 15–59 were eligible to be interviewed in the selected households. A total of 4,916 (out of 5,096) women and 4,568 (out of 4,769) men were interviewed yielding response rates of 97 percent and 96 percent respectively.

In the study, the dependent variable was exclusive breastfeeding. The following socio-demographic variables were used: mother’s age, marital status, education, region, mode of delivery, antenatal visits, place of delivery, infant’s size at birth, and infant’s sex. Some of the variables were recoded while others were adopted as reported in the 2008 GDHS. Ungrouped ages of mothers from 15 to 49 years old were coded as: 15–19, 20–35, or 36–49. Marital status of mothers was recoded as married, or not married. Level of education was also grouped into three categories (no education, primary, secondary/higher). In terms of region, all the ten regions in the country were considered. Antenatal visits by a mother were reorganized into three groups (0, 1–3, 4 or more visits). The generic modes of delivery (normal or caesarean) were considered. In relation to place of delivery, options were regrouped into three categories namely home, government health facility, and private health facility. An infant’s size at birth was considered as large, average, or small, and an infant’s sex was grouped as reported in the 2008 GDHS - male, or female.

Univariate analysis was used to present variables using descriptive analytical methods (frequencies and percentages). Bivariate analysis was also applied on the data. This involved cross-tabulations and results were presented in the form of proportions. Unadjusted odds ratio (UOR) of variables and their confidence intervals (CI) ware used to determine the presence of association between predictor variables and exclusive breastfeeding. Further, binary logistic regression was applied to examine the likely predictors of exclusive breastfeeding in the country. This method was deemed appropriate since binary logistic regression is a type of predictive model that can be used when the target or dependent variable is a categorical variable with two categories. With the present study, the dependent variable was categorized as either a mother was exclusively breastfeeding (code as *1*), or not exclusively breastfeeding (coded as *0*). All co-variates were included in generating regression model and the results were presented using adjusted odds ratio, and confidence intervals. In this work, 95% CI was used to test for statistical significance if the lower CI does not overlap the null value (Odds Ratio = 1). Stata version 11 was used to analyze the data.

Approval/Authorization to use the data was given by Measure DHS (Demographic and Health Survey), downloaded from DHS Data Archive. ICF Macro, 11785 Beltsville Drive, Calverton, MD 20705. http://www.measuredhs.com. On June 29, 2011.

## Results

A sample of 316 infants aged 0–5 months old were considered for analysis. The results in Table 1 show the distribution of the selected socio-demographic characteristics of mothers (15–49 years old). The age distribution indicated that more mothers (44%) were in the 20–35 years cohort while 23 percent were between 36–49 years group. In terms of marital status, about nine out of ten mothers were married. The distribution also showed that 41% had secondary or tertiary education, and about 26% attained primary education. Furthermore, with the residence of mothers, about 17% resided in the Northern Region. Brong Ahafo Region had the smallest number of mothers (7%). In the present study, the rate of exclusive breastfeeding was 64 percent (Table 2). The rate of exclusive breastfeeding is 84% in the first month, decreasing to 45% in the fifth month.

**Table 1:** Socio-demographic characteristics of respondents (n = 316)

**Socio-demographic variables**	**n**	**%**
Mother’s age		
15-19	102	32.3
20-35	140	44.3
36-49	74	23.4
Marital status		
Not married	28	8.9
Married	288	91.1
Mother’s education *		
No education	104	33.0
Primary	81	25.7
Secondary/higher	130	41.3
Residence		
Urban	117	37.0
Rural	199	63.0
Region		
Western	34	10.8
Central	30	9.5
Greater accra	35	11.1
Volta	32	10.1
Eastern	24	7.6
Ashanti	35	11.1
Brong ahafo	22	7.0
Northern	53	16.8
Upper east	24	7.6
Upper west	27	8.5
Mode of delivery		
Normal delivery	297	94.0
Caesarean section	19	6.0
Antenatal visits *		
0	11	3.5
1-3	61	19.6
4+	240	76.9
Place of delivery		
Home	131	41.5
Government/public facility	154	48.7
Private/PPAG/others	31	9.8
Child’s size at birth		
Large	179	57.4
Average	94	30.1
Small	39	12.5
Child’s sex		
Male	167	52.9
Female	149	47.2

* n < 316 due to recoding of variables.

**Table 2:** Infant age distribution and prevalence of exclusive breastfeeding (weighted)

**Infant age**	**n (%)**	**Prevalence**
0	27	83.9
1	58	58.2
2	62	64.7
3	62	60.7
4	53	56.0
5	54	45.3
	316*	64.4 **

*Total number of infants.

**Prevalence rate of exclusive breastfeeding for all ages.

The mode of delivery distribution indicated that most mothers (94%) had a normal vaginal delivery. Table 1 further showed that more mothers (77%) visited antenatal centers four or more times, and a few (4%) did not attend antenatally. Almost half of mothers (49%) gave birth in a government health facility and 10% gave birth at a private health facility. More than half the infants were considered as large (57%) at the time of their birth and about 13% were perceived to be small in size at birth.

The variables that exhibited association with exclusive breastfeeding in the bivariate analysis adjusting for infant age were marital status (married mothers), region (Volta Region, and Upper West Region), and place of delivery (government health facility) (Table 3). All variables were considered for the multivariate analysis. Results from the multivariate analysis indicated that Volta Region, government health facility, and an average size of infant at birth were the determinants of exclusive breastfeeding. The odds were 11.2 times higher in mothers who resided in the Volta Region (OR = 11.19; 95% CI: 2.57, 48.61) to practice exclusive breastfeeding compared to mothers in the Western Region. Also, mothers who delivered at a government health facility had odds of 3.4 times higher of practicing exclusive breastfeeding compared to mothers who delivered at home (OR = 3.4; 95% CI: 1.75, 6.62). Likewise, the odds ratio for exclusive breastfeeding was twice as high for mothers who perceived their infants to be an average size at birth compared to mothers whose infants were perceived to be large at birth (OR = 2.22; 95% CI: 1.14, 4.30).

**Table 3:** Logistic regression of determinants of exclusive breastfeeding

**Variables**	**Exclusive breastfeeding**	**Age-adjusted**		**Multivariate**	
	**(n = 200) n (%)**	**UOR**	**95% CI**	**AOR**	**95% CI**
Mother’s age					
15-19	61 (62.8)	1		1	
20-35	92 (68.2)	1.26	0.73, 2.19	1.22	0.65, 2.41
36-49	47 (64.4)	1.07	0.57, 2.01	0.75	0.37, 1.71
Marital status					
Not married	12 (44.4)	1		1	
Married	188 (67.6)	2.61	1.17, 5.82	1.81	0.70, 4.58
Mother’s education					
No education	73 (72.3)	1		1	
Primary	49 (63.6)	0.67	0.35, 1.27	0.72	0.31, 1.69
Secondary/higher	77 (61.1)	0.60	0.34, 1.06	0.44	0.19, 1.02
Region					
Western	17 (51.5)	1		1	
Central	17 (58.6)	1.33	0.49, 3.65	1.53	0.50, 4.64
Greater accra	20 (60.6)	1.45	0.54, 3.85	1.52	0.49, 4.70
Volta	28 (90.3)	8.78	2.22, 34.7	11.19	2.57, 48.61
Eastern	15 (65.2)	1.76	0.59, 5.29	2.19	0.64, 7.51
Ashanti	15 (44.1)	0.74	0.28, 1.95	0.69	0.24, 2.02
Brong ahafo	12 (57.1)	1.25	0.42, 3.78	1.08	0.32, 3.65
Northern	37 (72.6)	2.49	0.99, 6.24	2.95	0.92, 9.45
Upper east	18 (75.0)	2.82	0.89, 8.92	2.32	0.61, 8.79
Upper west	21 (80.8)	3.95	1.20, 13.03	3.48	0.91, 13.18
Antenatal visits					
0	6 (60.6)	1		1	
1-3	35 (60.3)	1.01	0.26, 4.00	0.96	0.21, 4.29
4+	157 (67.6)	1.36	0.37, 4.97	1.12	0.26, 4.84
Mode of delivery					
Normal	187 (65.4)	1		1	
Caesarean	13 (68.4)	1.15	0.42, 3.12	1.02	0.30, 3.44
Place of delivery					
Home	71 (57.3)	1		1	
Government facility	112 (74.7)	2.20	1.32, 3.67	3.41	1.75, 6.62
Private facility	17 (54.8)	0.91	0.41, 2.00	1.87	0.69, 5.02
Size of infant at birth					
Large	108 (62.1)	1		1	
Average	62 (68.9)	1.35	0.79, 2.33	2.22	1.14, 4.30
Small	28 (73.7)	1.71	0.78, 3.75	1.67	0.66, 4.22
Sex of infant					
Male	108 (66.7)	1		1	
Female	92 (64.3)	0.90	0.56, 1.45	0.96	0.56, 1.66

n (%): proportion per category; UOR: Unadjusted odds ratio; AOR: Adjusted odds ratio; CI: confidence interval.

## Discussion

The present study reported the prevalence of exclusive breastfeeding (6 months) in the country to be 64% as compared to 63% reported in the 2008 GDHS. The slight difference between the 2008 GDHS rate and the present study can be attributed to the sample size used. However, the sample size (n = 316 of infants 0–5 months old) can be verified using the individual (women) dataset. Notwithstanding the slight difference between the sample sizes the findings of this study can be validated.

The study analysis was based on the exclusive breastfeeding definition recommended by World Health Organization [8] as a mother or caregiver giving nothing else but breast milk to an infant in the last 24 hours preceding a survey. Based on this definition, the study identified three factors to be associated with exclusive breastfeeding in Ghana. These factors were mothers residing in Volta Region, delivery in a government health facility, and perceived average size of infant at birth.

In the present study, mothers who resided in the Volta Region were more likely to practice exclusive breastfeeding as compared to mothers in other regions of Ghana. The low practice of exclusive breastfeeding in all regions of Ghana can be attributed to cultural beliefs. Mothers or relatives usually give water and other concoctions to infants as a perceived way of quenching their thirst or as a sign of welcoming them into the world [5,9]. These cultural practices may be practiced less in the Volta Region. Hence, mothers in that region tend to be more likely to practice exclusive breastfeeding.

Furthermore, the study found that mothers who delivered at a government health facility had a higher probability to practice exclusive breastfeeding compared to mothers who delivered at home, or a private health facility. Place of delivery has been found in a number of studies to be associated with exclusive breastfeeding [10,11]. In the present study, delivery at a government health facility was identified as a predictor of exclusive breastfeeding and this conforms to studies in Ghana [12], and in Nigeria [13]. Delivery at hospital as a predictor can be attributed to the call made by WHO and UNICEF [1] for hospitals to be centers of breastfeeding. Since the introduction of the BFHI in Ghana, the rate of exclusive breastfeeding increased from about 2% in 1993 to 63% in 2008 [4]. This initiative, undoubtedly, might have accounted for government health facility being a predictor of exclusive breastfeeding in the country.

Also, mothers whose infants were perceived to be average in size at birth commonly practiced exclusive breastfeeding compared to mothers who perceived their infants to be large at birth. Mothers who perceive their infants to be large may be complacent to practice exclusive breastfeeding while those infants perceived to be small may lack the energy to suckle or may be preterm babies. As explained by Flaherman and colleagues, smaller newborns are more likely to receive formula [14], and other infusions to stabilize their condition. However, mothers who perceive their infants’ size to be of an average size may be most likely to practice exclusive breastfeeding for their infants to attain the expected body size faster; since such infants would have the strength to suckle more.

This study encountered a number of limitations. The sample size used in this study was small and that could have contributed to only a couple of variables been statistically associated with the practice of exclusive breastfeeding in the multivariate analysis. The cross-sectional approach adopted for this study limits its ability to draw inferential causalities from the predictor variables and the practice of exclusive breastfeeding in the country.

## Conclusions

Exclusive breastfeeding has been accepted as an essential practice that promotes healthy growth and development of an infant, and subsequently confers physiological benefits to the mother. Mothers in the Volta Region, those who delivered at a government health facility, and infants perceived to be average in size at birth were found to be associated with exclusive breastfeeding in Ghana. It is therefore recommended that Information, Education and Communication (IEC) programmes targeting mothers who are less likely to practice exclusive breastfeeding be formulated, implemented, and monitored accordingly by the Ministry of Health. This will facilitate in the achievement of the fourth Millennium Development Goal in Ghana.

## Competing interests

Both authors declare that they have no competing interests.

## Authors’ contributions

AMTN participated in acquiring the data and performed the statistical analysis. AMTN and AKK helped to draft the manuscript. Both authors read and approved the final manuscript.
